# No evidence for association of *MTHFR* 677C>T and 1298A>C variants with placental DNA methylation

**DOI:** 10.1186/s13148-018-0468-1

**Published:** 2018-03-13

**Authors:** Giulia F. Del Gobbo, E. Magda Price, Courtney W. Hanna, Wendy P. Robinson

**Affiliations:** 1 BC Children’s Hospital Research Institute, 950 W 28th Ave, Vancouver, BC V5Z 4H4 Canada; 2 Department of Medical Genetics, University of British Columbia, 4500 Oak St, Vancouver, BC V6H 3N1 Canada; 3 Epigenetics Programme, Babraham Institute, Cambridge, CB22 3AT UK; 4 Centre for Trophoblast Research, University of Cambridge, Cambridge, CB2 3EG UK; 5 Child and Family Research Institute, Room 2082, 950 W 28th Avenue, Vancouver, BC V5Z 4H4 Canada

**Keywords:** MTHFR, DNA methylation, Placenta, One-carbon metabolism, 450k array, Neural tube defects, Preeclampsia, IUGR

## Abstract

**Background:**

5,10-Methylenetetrahydrofolate reductase (MTHFR) is a key enzyme in one-carbon metabolism that ensures the availability of methyl groups for methylation reactions. Two single-nucleotide polymorphisms (SNPs) in the *MTHFR* gene, 677C>T and 1298A>C, result in a thermolabile enzyme with reduced function. These variants, in both the maternal and/or fetal genes, have been associated with pregnancy complications including miscarriage, neural tube defects (NTDs), and preeclampsia (PE), perhaps due to altered capacity for DNA methylation (DNAm). In this study, we assessed the association between *MTHFR* 677TT and 1298CC genotypes and risk of NTDs, PE, or normotensive intrauterine growth restriction (nIUGR). Additionally, we assessed whether these high-risk genotypes are associated with altered DNAm in the placenta.

**Results:**

In 303 placentas screened for this study, we observed no significant association between the occurrence of NTDs (*N* = 55), PE (early-onset: *N* = 28, late-onset: *N* = 20), or nIUGR (*N* = 21) and placental (fetal) *MTHFR* 677TT or 1298CC genotypes compared to healthy pregnancies (*N* = 179), though a trend of increased 677TT genotype in PE/IUGR together was observed (OR 2.53, *p* = 0.048). DNAm was profiled in 10 high-risk 677 (677TT + 1298AA), 10 high-risk 1298 (677CC + 1298CC), and 10 reference (677CC + 1298AA) genotype placentas. Linear modeling identified no significantly differentially methylated sites between high-risk 677 or 1298 and reference placentas at a false discovery rate < 0.05 and Δβ ≥ 0.05 using the Illumina Infinium HumanMethylation450 BeadChip. Using a differentially methylated region analysis or separating cytosine-guanine dinucleotides (CpGs) by CpG density to reduce multiple comparisons also did not identify differential methylation. Additionally, there was no consistent evidence for altered methylation of repetitive DNA between high-risk and reference placentas.

**Conclusions:**

We conclude that large-scale, genome-wide disruption in DNAm does not occur in placentas with the high-risk *MTHFR* 677TT or 1298CC genotypes. Furthermore, there was no evidence for an association of the 1298CC genotype and only a tendency to higher 677TT in pregnancy complications of PE/IUGR. This may be due to small sample sizes or folate repletion in our Canadian population attenuating effects of the high-risk *MTHFR* variants. However, given our results and the conflicting results in the literature, investigations into alternative mechanisms that may explain the link between *MTHFR* variants and pregnancy complications, or in populations at risk of folate deficiencies, are warranted.

**Electronic supplementary material:**

The online version of this article (10.1186/s13148-018-0468-1) contains supplementary material, which is available to authorized users.

## Background

One-carbon metabolism (OCM) is a fundamental biochemical pathway that activates and transfers methyl (CH_3_) groups for purine synthesis and methylation of DNA, proteins, and lipids, making it important for processes such as DNA synthesis, cellular division, and proliferation. Both functional and dietary deficiencies are thought to contribute to altered OCM cycling. Several B vitamins act as substrates or cofactors for OCM, most notably vitamin B_9_ or folate, the transporter of methyl groups in OCM. Genetic variants in a central OCM enzyme, 5,10-methylenetetrahydrofolate reductase (MTHFR), have been heavily researched in association with human diseases, such as cardiovascular disease, pregnancy complications, and cancers [1–4]. MTHFR catalyzes the irreversible reduction of 5,10-methylenetetrahydrofolate (5,10-CH_3_-THF) to 5-methyltetrahydrofolate (5-CH_3_-THF). 5-CH_3_-THF is subsequently used as the substrate for the conversion of homocysteine to methionine, catalyzed by the enzyme methionine reductase. Methionine is then used to synthesize *S*-adenosylmethionine (SAM), the universal methyl donor for methylation reactions, including DNA methylation (DNAm), catalyzed by DNA methyltransferases (DNMTs). As such, MTHFR is key to directing one-carbon units toward DNAm reactions, which has motivated the investigation of alterations in DNAm as the mechanism underlying the association of genetic variants in *MTHFR* with various pathologies.

Two single-nucleotide polymorphisms (SNPs) in the *MTHFR* gene, 677C>T (rs1801133) and 1298A>C (rs1801131), result in reduced MTHFR function in vitro, particularly in the homozygous recessive state [5–8]. These variants are common in the population; globally, the variant allele frequencies are approximately 0.25–0.3 (dbSNP [9]), though frequencies vary between different populations. These variants have been associated with markers of altered OCM, such as increased levels of homocysteine and altered levels of blood folates [10–16], most consistently for the 677 variant. High-risk *MTHFR* genotypes (677TT and 1298CC) or variant alleles (677T and 1298C) have been found in association with a number of reproductive and developmental pathologies. *MTHFR* 677 and 1298 variants in affected pregnancies or parents have been associated with miscarriage [17–19] and neural tube defects [20–25]. The 677T allele and 677TT genotype in mothers have been associated with preeclampsia (PE), a maternal hypertensive disorder in pregnancy [26–28]. Associations between fetal-placental *MTHFR* 677TT genotypes have been identified [29], though these are not as well studied as the maternal variants.

Researchers have hypothesized that increased risk of pathology might be attributed to aberrant patterns in DNAm, resulting from altered OCM flux caused by these *MTHFR* variants [25, 30, 31]. While several studies have investigated the association of the *MTHFR* 677C>T and 1298A>C variants with altered DNAm, results are inconsistent; some have reported associations between the high-risk homozygous *MTHFR* genotypes and/or folate levels and altered DNAm [32–37], whereas others find no association [38–41]. As gene expression, DNAm patterns, and metabolic requirements are highly variable between tissues, even these conflicting results ascertained in adult, non-pregnant blood, may not generalize to pregnancy complications.

The placenta is a directly relevant tissue in which to study the interaction between *MTHFR* variants, altered DNAm, and pregnancy complications. Due to the demand for DNA synthesis, cellular division, and proliferation by the growing fetus and placenta, the requirement for folate during pregnancy increases by approximately 5–10 times the level of non-pregnant women [42]. High-affinity folate receptors on maternal-facing trophoblast cells allow the placenta to transport and concentrate folate from the maternal blood up to three times within the placenta [43, 44], ensuring the availability of this crucial nutrient during development. Consistent with studies in other tissues, the *MTHFR* 677T allele is associated with reduced MTHFR enzyme function in the placenta [45]. If OCM flux is impaired and DNAm patterns are altered in the placenta due to reduced variant MTHFR function, this could have implications for placental function and thus increase risk of pregnancy complications. Aberrant DNAm in the form of imprinting is known to have significant impact on placental development (reviewed in [46, 47]). Genome-wide or imprinted gene-specific alterations in DNAm have been noted in placental insufficiency complications of intrauterine growth restriction (IUGR) [48] and in early-onset PE [49–51]. Additionally, the placenta is a tissue that may be more likely to exhibit altered DNAm in response to reduced MTHFR enzyme function. The placenta exhibits a high degree of within- and between-individual variability in DNAm [52, 53], suggesting that it may be tolerant to changes in DNAm, allowing this organ to adapt to environmental conditions [52–54].

To date, no studies have investigated the association between DNAm in the placenta and the high-risk *MTHFR* 677TT and 1298CC genotypes. This may provide insight in to how the *MTHFR* variants have been previously associated with pregnancy complications, and potentially help to resolve the currently conflicting literature investigating the association between these variants and DNAm in other tissues. In this study, we evaluated whether fetal high-risk *MTHFR* genotypes were more prevalent in pregnancy complications of PE, IUGR, and neural tube defects (NTDs) using 303 placental DNA samples. The DNAm patterns of 30 placentas were heavily profiled using both site-specific and genome-wide techniques, including the Illumina Infinium HumanMethylation450 BeadChip array and repetitive DNA methylation, to understand the relationship between high-risk *MTHFR* 677TT and 1298CC genotypes and DNAm in the placenta.

## Methods

### Ethics and sample collection

Ethics approval for this study was obtained from the University of British Columbia/Children’s Hospital and Women’s Health Centre of British Columbia Research Ethics Board (H04-70488, H10-01028). Placentas were collected from term deliveries at BC Women’s Hospital and Health Centre and from second trimester stillbirths, elective terminations, and spontaneous abortions through the embryo-fetal pathology laboratory. Cases with a prenatally identified chromosomal abnormality were excluded.

A minimum of two distinct sites were sampled from the fetal side of each placenta after fetal membranes (amnion and chorion) were removed. Samples were washed thoroughly with PBS to remove maternal blood. DNA was extracted by a standard salting-out procedure modified from Miller et al. [55] and quality evaluated using a NanoDrop ND-1000 (Thermo Scientific). One site from each placenta was selected at random for genotyping. As DNAm varies significantly within the placenta [52, 53], DNA was combined in equal amounts from at least two sites to generate a more representative sample in which to evaluate placental DNAm.

### Case characteristics

A total of 303 placentas were screened for *MTHFR* 677 and 1298 polymorphisms. These included 179 placentas from uncomplicated pregnancies, 48 from pregnancies associated with PE (28 early-onset PE, 20 late-onset PE), 21 from pregnancies associated with intrauterine growth restriction in the absence of maternal hypertension (normotensive IUGR, nIUGR), and 55 from pregnancies with a fetal NTD (Table 1). PE was defined according to the Society of Obstetricians and Gynaecologists of Canada (SOGC) criteria as pregnancies with (i) gestational hypertension (BP > 140/90 mmHg) and proteinuria (> 300 g/day) arising after 20 weeks of gestation; (ii) pre-existing hypertension with superimposed gestational hypertension, proteinuria, and/or one or more adverse maternal or fetal conditions; or (iii) gestational hypertension without proteinuria, with one or more adverse maternal or fetal conditions [56]. PE was subdivided into early-onset preeclampsia (EOPE), defined as a diagnosis of PE before 34 weeks of gestation, and late-onset preeclampsia (LOPE), a diagnosis of PE after 34 weeks of gestation [57]. IUGR commonly co-occurs with PE and was also defined following the SOGC criteria as birth weight < 3rd percentile, accounting for both fetal sex and gestational age (GA), or birth weight < 10th percentile with additional clinical findings indicative of poor growth such as uterine artery notching, absent or reversed end-diastolic velocity on Doppler ultrasound, or oligohydramnios [58]. nIUGR was defined as unexplained IUGR without the presence of maternal hypertension. NTDs were defined as a fetus diagnosed with spina bifida, anencephaly, or encephalocele on ultrasound and/or fetal autopsy.

**Table 1:** Clinical characteristics of cases

	*N*	Sex; *N* male (% male)	Gestational age (weeks); median (range)	Maternal age (years); median (range)
Control	179	85 (47)	39.6 (19.4–41.9)	34.4 (23.8–42.7)
EOPE	28	17 (61)	32.7 (23.6–38.4)*	36.0 (19.7–42.9)
LOPE	20	10 (50)	38.5 (34.9–41.4)*	34.3 (26.1–41.5)
nIUGR	21	9 (42)	36.2 (24.0–40.6)*	34.5 (26.1–42.8)
NTD	55	28 (51)	21.0 (16.7–23.7)*	30.4 (17.7–40.6)*

*EOPE* early-onset preeclampsia, *LOPE* late-onset preeclampsia, *nIUGR* normotensive intrauterine growth restriction, *NTD* neural tube defect

*p < 0.05, calculated in comparison to the control group by Fisher’s exact test for categorical variables and Mann-Whitney test for continuous variables

### *MTHFR* genotyping

Placental DNA was genotyped for the *MTHFR* 677 and 1298 polymorphisms using pyrosequencing. Primer sequences and reaction conditions can be found in Additional file 1: Table S1. Five microliters of PCR product was sequenced on a PyroMark Q96 MD Pyrosequencer (Qiagen) using standard protocols [59]. A subset of the genotyping results from the NTD group (*N* = 36) has been published elsewhere [60].

### Population stratification

Minor allele frequencies for the *MTHFR* 677 and 1298 SNPs vary significantly between different populations [61–64], as do the prevalence of NTDs and PE/IUGR pathologies [65]. Both high-risk *MTHFR* genotypes vary by ethnicity and geography, indicating that selective pressures have influenced their frequencies [62, 66]. Therefore, prior to performing a genetic association analysis, we aimed to assess whether our pregnancy complication groups were matched for ancestry. Maternal self-reported ethnicity was available for only 67% of cases, and no information about the father’s ethnicity was available. We thus used a panel of 57 ancestry-informative marker (AIM) SNPs [67–69] that were developed to distinguish between African, European, East Asian, and South Asian ancestries to infer the ancestry of study samples and assess population stratification along three major axes of variation.

Two hundred seventy-seven placental villus DNA samples were successfully genotyped at 53 AIMs using the Sequenom iPlex Gold platform by the Génome Québec Innovation Centre at McGill University, Montréal, Canada, with a call rate > 0.9 for both SNPs and samples. Multidimensional scaling (MDS) with *k* = 3 dimensions was performed in our study samples (*N* = 277) in addition to individuals (*N* = 2157) from African, East Asian, European, and South Asian populations from the 1000 Genomes Project (1kGP) [64] using 50 of the AIM genotypes that were available in both cohorts. This method allows 1kGP samples to be used as ancestry reference populations for our admixed population and has been used to identify ancestry outliers [70, 71]. The first three MDS coordinates were extracted for each sample and used to describe ancestry along a continuum rather than in discrete groups. We believe this better reflects ancestry in admixed populations such as that in Vancouver, as well as potentially better representing variation within an ancestry group. Further description of this method is included in Additional file 2: Methods.

### *MTHFR* genotype and DNAm

To assess the association of *MTHFR* genotype with DNAm, a subset of 30 control/mild pregnancy complication placentas were selected for in-depth DNAm profiling, hereafter referred to as the placental DNAm samples. None of these placentas had chromosomal abnormalities, as confirmed by multiplex ligation-dependent probe amplification in one or more sites per placenta. The effect of each *MTHFR* SNP was assessed independently by comparing 10 placentas with reference genotype at each *MTHFR* SNP (677CC + 1298AA), 10 placentas with the high-risk 677TT genotype in combination with the reference 1298AA (termed “high-risk 677”), and 10 placentas with high-risk 1298CC genotype in combination with the reference 677CC genotype (termed “high-risk 1298”). The reference, high-risk 677, and high-risk 1298 groups were matched by sex, gestational age, birth weight, and maternal reported ethnicity (Table 2). To obtain sufficient numbers in each genotype group, as the high-risk genotypes are relatively rare in our population and we additionally excluded heterozygotes at either locus, some mild pregnancy complication cases (LOPE without IUGR and nIUGR) were included. We previously found no evidence for altered placental DNAm associated with these phenotypes compared to controls in a separate study [51].

**Table 2:** Clinical characteristics of placental DNAm samples

	*N*	Sex; *N* male (%)	Gestational age (weeks); median (range)	Maternal ethnicity; *N* Caucasian (%)	Birth weight (SD); median (range)
Reference (677CC + 1298AA)	10	4 (40)	39.0 (36.1–41.6)	8 (80 %)	0.055 (− 1.13–1.40)
High-risk 677 (677TT + 1298AA)	10	4 (40)	37.9 (34.6–40.3)	6 (60 %)	− 0.13 (− 2.97–0.70)
High-risk 1298 (677CC + 1298CC)	10	5 (50)	39.4 (38.6–40.7)	8 (80 %)	0.005 (− 1.61–2.20)

*p* values were calculated in comparison to the reference group by Fisher’s exact test for categorical variables and Mann-Whitney test for continuous variables

*SD* standard deviation

### Illumina Infinium HumanMethylation450 BeadChip

Combined placental DNA from the 30 placental DNAm samples described in Table 2 was purified using the Qiagen DNeasy Blood and Tissue kit, and 750 ng of this DNA was bisulfite converted using the EZ DNA Methylation kit (Zymo Research) following the manufacturer’s protocols. Samples were processed following the Illumina Infinium HumanMethylation450 BeadChip (450k array) protocol [72] and scanned using the Illumina HiScan 2000. Raw intensity was read into Illumina Genome Studio software 2011.1, and background normalization was applied. Data processing was performed as described in Price et al. [73], including sample quality checks, probe filtering, data normalization, and batch correction. This processing pipeline resulted in a final *N* = 442,355 cytosine-guanine dinucleotide (CpG) sites from the 450k array for analysis.

### Repetitive DNA methylation

In addition to the 450k array, genome-wide DNAm was also assessed using DNAm at repetitive Alu, LINE-1, and ribosomal DNA (rDNA) sequences [74] by pyrosequencing in the 30 placental DNAm samples. These sequences are dispersed throughout the genome, allowing DNAm to be measured at many sites using a single assay per repetitive sequence. DNAm at these three repetitive DNA sequences has been shown to be altered due to different environmental or biological factors [75–79]. Three hundred nanograms of purified combined placental villi DNA was bisulfite converted using the EZ DNA Methylation-Gold Kit (Zymo Research) following the manufacturer’s protocol. Alu and LINE-1 elements were amplified using primer sets designed to complement the L1H and AluSx consensus sequences [80], and rDNA repeats were amplified using primers designed to target the rDNA promoter [81] (Additional file 1: Table S1). PCR products were sequenced on a PyroMark Q96 MD Pyrosequencer (Qiagen) using standard protocols [59]. The DNAm status of each CpG dinucleotide (Alu: *N* = 3; LINE-1: *N* = 4; rDNA: *N* = 26) was evaluated using the PyroQ CpG software (Biotage). For each assay, the correlation of DNAm between CpGs was confirmed, and then an average DNAm was calculated across the CpGs within each assay in each sample.

### Statistical analyses

Statistical analyses were conducted in R statistical software [82]. Deviation from Hardy-Weinberg equilibrium (HWE) at the two *MTHFR* SNPs in controls was assessed using an exact test for HWE. Differences in the distribution of ancestry MDS coordinate values between control and pregnancy complication groups (EOPE, LOPE, nIUGR, NTD) were assessed using the Kolmogorov-Smirnov test. The association between the frequency of *MTHFR* 677TT and/or 1298CC genotypes and pregnancy complications was assessed using a one-tailed Fisher’s exact test to test the hypothesis that the high-risk genotypes will be increased in pregnancy complications compared to controls. For the placental DNAm samples, 450k array-wide average DNAm and percent outlier probes per sample were calculated as in Price et al. [60]. Altered measures of genome-wide methylation, including 450k array-wide average, percent outlier probes, Alu, LINE-1, and rDNA methylation, were assessed using the Mann-Whitney test. All *p* values from statistical tests involving multiple comparisons (ancestry MDS coordinate values, altered genome-wide DNAm measures) were corrected for multiple testing using the Bonferroni method.

450k array site-specific differential methylation was also assessed as in Price et al. [60]. Briefly, a linear model with the *MTHFR* group as the main effect and fetal sex and gestational age included as covariates was fit to every CpG on the array (*N* = 442,355). Differential methylation results were then extracted for the comparison of high-risk 677 to reference and for that of high-risk 1298 to reference. These comparisons were used to calculate group differences in DNAm (delta-beta, Δβ). Significantly differentially methylated CpG sites were considered as those with a false discovery rate (FDR) < 0.05 and Δβ ≥ 0.05. Two dimension-reduction techniques were additionally used in the 450k array data: a differentially methylated region (DMR) analysis, as in Price et al. [60], and an assessment of differential DNAm depending on the CpG density of the surrounding region. 450k probes were separated into four groups based on the CpG density: high-density islands, island shores, intermediate-density islands, and non-islands, defined as per Price et al. [83], and the unadjusted *p* value distributions from the linear model were assessed in each CpG density group separately.

## Results

### Analysis of ancestry-informative markers identifies no significant population stratification

**Fig. 1:**
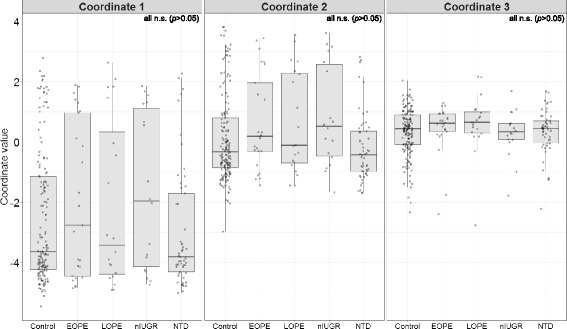
Distribution of ancestry derived from multidimensional scaling of AIM genotypes in control and pregnancy complication placentas. In *N* = 28 EOPE, *N* = 20 LOPE, *N* = 21 nIUGR, or *N* = 55 NTD placentas, there were no significant differences in the distribution of ancestry MDS coordinate values compared to *N* = 179 controls at either of the three ancestry MDS coordinates (Kolmogorov-Smirnov tests, Bonferroni-corrected *p* > 0.05). This suggests that there is no population stratification by ancestry in the groups selected for this study. EOPE early-onset preeclampsia, LOPE late-onset preeclampsia, nIUGR normotensive intrauterine growth restriction, NTD neural tube defect

Prior to testing for the association of fetal *MTHFR* genotypes with NTDs or PE/IUGR groups, we sought to confirm that these pregnancy complication groups were not confounded with ancestry as the frequency of the *MTHFR* 677 and 1298 variants varies between different ancestry groups [61–63]. Self-reported ethnicity was available from mothers for only 67% (203/303) of samples, which were predominately of European (Caucasian) and East Asian ancestries. We thus described ancestry using coordinates 1, 2, and 3 obtained through MDS of genotypes at 50 AIMs (Additional file 3: Table S2) in 277 of our placental samples along with 2157 samples from the 1kGP [64] (Additional file 4: Figure S1). These three MDS coordinates were significantly different between the four major continental populations from 1kGP (Additional file 4: Figure S1). Furthermore, for those samples for which we had both maternal reported ethnicities in addition to AIMs (*N* = 181), the three ancestry MDS coordinates were highly concordant with maternal reported ethnicity (Additional file 5: Figure S2). These findings suggest that this method is adequate to describe major patterns of genetic ancestry. There was no significant difference in the distribution of ancestry MDS coordinate values 1, 2, and 3 between the NTD, PE, and nIUGR pathology groups in comparison to controls (Fig. 1). We thus concluded that our pathology groups do not show evidence of confounding by ancestry.

### *MTHFR* 677TT and 1298CC genotypes are not significantly associated with PE or NTDs

To investigate whether the *MTHFR* 677TT and 1298CC genotypes were associated with PE, nIUGR, or NTD pathologies, we genotyped placentas at these two loci from 179 control, 28 EOPE, 20 LOPE, 21 nIUGR, and 55 NTD pregnancies. Neither SNP deviated from HWE in controls (Additional file 6: Table S3). In our population of 303 placentas collected in Vancouver, Canada, the frequencies of the variant *MTHFR* 677T and 1298C alleles were 0.295 and 0.290, respectively. There was no significant increase in the frequency of the high-risk *MTHFR* 677TT or 1298CC genotypes in EOPE, LOPE, nIUGR, or NTD cases compared to controls (Table 3). There was, however, a tendency for increased *MTHFR* 677TT genotype in placentas from pregnancies complicated by placental pathologies (PE or nIUGR). When considered together (PE or nIUGR; *N* = 69), the increase in *MTHFR* 677TT frequency compared to controls was nominally significant (OR = 2.53, *p* = 0.048).

**Table 3:** *MTHFR* 677TT and 1298CC genotypes in pregnancy complications

	*N*	677TT frequency (*N*)	*p* value^†^	OR (95% CI)	1298CC frequency (*N*)	*p* value^†^	OR (95% CI)
Control	179	0.056 (10)	–	–	0.101 (18)	–	–
EOPE	28	0.107 (3)	0.249	2.02 (0.33–8.59)	0 (0)	1.00	0
LOPE	20	0.150 (3)	0.129	2.96 (0.48–13.1)	0.150 (3)	0.355	1.57 (0.27–6.27)
nIUGR	21	0.143 (3)	0.143	2.80 (0.45–12.3)	0.048 (1)	0.891	0.449 (0.01–3.16)
NTD	55	0.091 (5)	0.260	1.69 (0.43–5.72)	0.073 (4)	0.809	0.70 (0.16–2.27)

*OR* odds ratio, *CI* confidence intervals, *EOPE* early-onset preeclampsia, *LOPE* late-onset preeclampsia, *nIUGR* normotensive intrauterine growth restriction, *NTD* neural tube defect

^†^*p* values, calculated by one-tailed Fisher’s exact test

### High-risk *MTHFR* 677 and 1298 genotypes are not associated with altered genome-wide DNAm in the placenta

Due to the central role that MTHFR plays in OCM, the high-risk *MTHFR* genotypes are often hypothesized to affect the cell’s ability to methylate DNA. We anticipated that such effects could potentially be more pronounced in the placenta due to its high demand for folate in pregnancy. We selected a subset of 30 placental samples with no, or mild, pathology in which to profile DNAm using both genome-wide and site-specific approaches. The selected samples were of three *MTHFR* genotype groups: (1) reference (*N* = 10; *MTHFR* 677CC + 1298AA), (2) high-risk 677 (*N* = 10; *MTHFR* 677TT + 1298AA), and (3) high-risk 1298 (*N* = 10; *MTHFR* 677CC + 1298CC). No cases with high-risk genotypes at both loci were available in our population to test.

First, these 30 placental DNAm samples were run on the 450k array, from which several measures of DNAm were obtained. Array-wide DNAm was calculated by averaging of 442,355 CpG sites in each sample. This array-wide measure of DNAm did not differ significantly between either of the high-risk *MTHFR* groups and the reference group (Table 4). Altered genome-wide DNAm might not be a characteristic of all carriers of the *MTHFR* variants; thus, we also calculated the percentage of outlier CpG sites from the 450k array for each sample to identify individuals exhibiting outlying patterns of DNAm [84]. Though there was no significant difference in outlier CpGs between the high-risk 677 and reference group (Table 4), there was a trend for more outlying CpG sites in the high-risk 1298 group than in the reference (Table 4, Bonferroni-corrected *p* = 0.058).

**Table 4:** Genome-wide measures of altered DNAm in high-risk *MTHFR* and reference placentas

	Array-wide average DNAm (*β*)	Outlier array sites (%)	Alu DNAm (%)	LINE-1 DNAm (%)	rDNAm (%)
Reference (*N* = 10)	0.407 (0.396–0.413)	0.661 (0.262–0.993)	18.9 (17.6–21.7)	52.8 (51.0–53.8)	19.4 (11.2–30.9)
High-risk 677 (*N* = 10)	0.405 (0.397–0.409)	0.851 (0.262–6.357)	20.1 (15.7–21.4)	53.5 (49.8–57.6)	20.0 (8.4–28.9)
High-risk 1298 (*N* = 10)	0.405 (0.395–0.413)	1.14 (0.501–3.37)	19.8 (17.5–21.6)	51.0 (46.5–55.0)	22.9 (10.2–28.9)

All results are reported as median (range); *p* values were calculated by the Mann-Whitney test for the comparison of high-risk 677 or high-risk 1298 to the reference group with Bonferroni correction for multiple comparisons

*β* beta value, *rDNAm* ribosomal RNA genes

Next, the methylation of repetitive DNA sequences was assessed in the 30 placental DNAm samples. Repetitive DNAm assays target numerous sites in the genome that are not well covered by the 450k array, and thus give an additional measure of genome-wide DNAm. No significant alterations in the DNAm of Alu, LINE-1, or rDNA sequences were identified between either of the high-risk *MTHFR* genotype groups and the reference genotype group (Table 4). Slightly higher methylation was seen for the high-risk 677 group in all comparisons, though the range of values was similar. There was, however, a trend for decreased LINE-1 DNAm in the high-risk 1298 group compared to the reference group (nominal *p* = 0.052), but this is not meaningful after correction for multiple comparisons. Overall, we find no conclusive evidence for altered genome-wide DNAm in association with the high-risk 677 or high-risk 1298 genotypes in the placenta using these DNAm measures.

### High-risk *MTHFR* 677 and 1298 genotypes are not associated with altered site-specific DNAm in the placenta

**Fig. 2:**
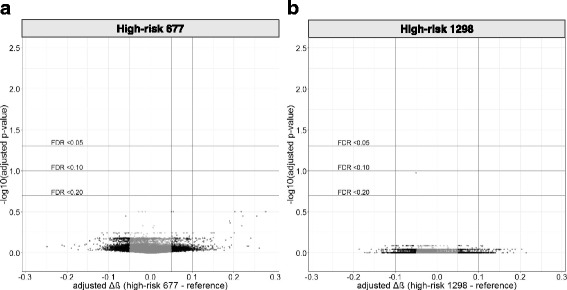
450k array-wide differential DNAm volcano plots in high-risk *MTHFR* 677 and high-risk 1298 placentas. Differential methylation was determined using a linear model with the *MTHFR* group as the main effect and fetal sex and gestational age included as covariates. The magnitude of difference (Δβ) between risk groups and reference group is depicted on the *x*-axis, and the significance of the comparison (−log10(adjusted *p* value)) is on the *y*-axis, for every CpG tested on the 450k array (*N* = 442,355). **a** Differential methylation results between high-risk 677 and reference placentas. **b** Differential methylation results between high-risk 1298 and reference placentas. Neither comparison identified any CpG sites differentially methylated between the high-risk *MTHFR* placentas and reference placentas. 450k array Illumina Infinium HumanMethylation450 BeadChip, FDR false discovery rate

The DNAm status of individual CpG sites targeted by the 450k array in association with the high-risk *MTHFR* genotype groups was next assessed. A linear model was fit to each CpG site to test for differential methylation by the genotype group, including sex and gestational age at birth as covariates. None of the 442,355 CpG sites was differentially methylated at a FDR < 0.05 and methylation difference (Δβ) > 0.05 in either of the high-risk *MTHFR* genotype groups compared to the reference group (Fig. 2).

Following this finding, two dimension-reduction techniques were utilized to explore whether identification of differences between high-risk *MTHFR* groups and controls in the 450k array data was limited due to a small sample size or large number of test sites. Due to structural or functional differences, some genomic regions may be more vulnerable to the effects of a reduced ability to methylate DNA potentially caused by the presence of variant MTHFR enzymes. As such, 450k probes were separated into four groups based on the CpG density of the surrounding region: high-density islands, island shores, intermediate-density islands, and non-islands. Additionally, a DMR finding tool was utilized to identify whether any DMRs existed between high-risk *MTHFR* genotype placentas and controls. Unadjusted *p* value distributions did not show differential methylation at any of the four CpG density groups between high-risk *MTHFR* and reference placentas (Additional file 7: Figure S3), nor were any significant DMRs identified. Given these results, we conclude that large-scale alterations in DNAm at CpG sites measured by the 450k array in the placenta are not commonly associated with high-risk 677 or 1298 *MTHFR* genotypes in our population.

## Discussion

Altered DNAm has been proposed as a mechanism through which *MTHFR* 677TT and 1298CC genotypes have been associated with pregnancy complications and other pathologies [25, 30, 31]. In this study, we sought to investigate alterations in DNAm in association with high-risk *MTHFR* 677TT and 1298CC genotypes in the placenta, a crucial tissue for the development of the fetus and a healthy pregnancy. Despite deeply profiling DNAm in *N* = 10 high-risk 677, *N* = 10 high-risk 1298, and *N* = 10 reference placentas using a variety of measures, we identified no evidence for altered placental genome-wide or site-specific DNAm in association with high-risk *MTHFR* genetic variants.

Given the fundamental involvement of OCM in activating and transporting methyl units, if the variant *MTHFR* alleles influence DNAm, this effect is predicted to be widespread and not gene-specific [85]. By using the 450k array, we interrogated DNAm at over 440,000 sites in the placental genome, assessing specific CpG sites and also genome-wide trends. This array covers 99% of RefSeq genes and is widely dispersed across genomic features and, therefore, can provide an accurate reflection of genome-wide changes associated with specific genomic features or gene regulation. No significant differences in the numerous measures of altered 450k array genome-wide or site-specific DNAm were identified, despite additionally utilizing dimension-reduction techniques to account for a small sample size and large number of test sites. DNAm at repetitive DNA sequences, including Alu and LINE-1 repetitive elements and rDNA repeats, was also assessed, as they are not well covered by the array, and they allow us to interrogate numerous locations in the genome in one pyrosequencing assay. The Alu and LINE-1 repetitive elements have previously been used as surrogate measures for genome-wide DNAm [74, 86], and all three repetitive sequences have exhibited alterations in DNAm in certain pathologies and in response to environmental exposures [75–79]. Though small sample size limited our power to detect significant differences in DNAm in this study, we aimed to mitigate this by deeply profiling the 30 placental DNAm samples using a variety of measures of DNAm to assess whether any differential methylation exists in association with the high-risk *MTHFR* genotypes. Our study cannot fully exclude that subtle differences in placental DNAm exist in association with high-risk *MTHFR* genotypes or that a subset of at-risk placentas might show changes in DNAm while the groups as a whole did not. Despite this, the results from these numerous genome-wide assays reveal that at the very least, large magnitude and/or array-wide differential methylation does not commonly occur in association with high-risk *MTHFR* genotype in the placenta.

Our study is the first that has investigated the associations between *MTHFR* 677 and 1298 variants and altered DNAm in the placenta, and only the second that has done so using a genome-wide DNAm microarray platform. Numerous studies have investigated altered DNAm in association with *MTHFR* 677 and/or 1298 variants using different measures of genome-wide DNAm and/or targeted gene DNAm, summarized in Table 5. Results from these various studies, mainly in the blood, are conflicting. Certain studies have found associations between *MTHFR* 677 or 1298 polymorphisms and altered DNAm; however, many do not find significant associations with altered DNAm or only find altered DNAm in the presence of low levels of OCM nutrients (Table 5). Some of these inconsistencies may be explained by the use of different measures of altered DNAm (i.e., genome-wide, candidate site-specific, repetitive element DNAm) between studies, lack of multiple-test correction, use of different tissues, or inconsistencies in accounting for confounding variables. Nonetheless, the effect of the *MTHFR* 677 and 1298 variants on DNAm is clearly complex.

**Table 5:** Literature assessing associations between *MTHFR* 677 or 1298 variants and altered DNAm in healthy tissues

Study	Type of DNAm assessed: specific assay	Tissue	Study size^†^	Results
Studies finding associations with DNAm				
Stern et al. (2000) [32]	Genome-wide: radiolabeled methyl group incorporation assay	Blood	677CC: *N* = 9 677TT: *N* = 10	677TT associated with approximately 40% higher [^3^H]-methyl acceptance capacity than 677CC (*p* = 0.04), reflecting global hypomethylation
Castro et al. (2004) [34]	Genome-wide: cytosine extension assay	Blood	677CC/1298AA: *N* = 17 677CT/1298AA: *N* = 22 677TT/1298AA: *N* = 9 677CT/1298AC: *N* = 22 677CC/1298AC: *N* = 20 677CC/1298CC: *N* = 7	677TT associated with higher [^3^H]-dCTP relative incorporation compared to 677CC (*p* < 0.05). 677TT/1298AA and 677CC/1298CC associated with higher relative incorporation than 677CC/1298AA (*p* < 0.05)
McKay et al. (2012) [31]	Genome-wide: LUMA Candidate sites (*N* = 3): pyrosequencing	Umbilical cord blood	677: *N* = 160 1298: *N* = 132 mother-infant pairs	Maternal 677T allele associated with altered mean DNAm in *IGF2* in infant cord blood (*p* = 0.017); maternal 1298C allele associated with altered DNAm at 1 CpG in *ZNT5* (*p* = 0.012) in infant cord blood. No associations with genome-wide DNAm
van Mil et al. (2014) [100]	Candidate sites (*N* = 11): MassArray EpiTYPER	Umbilical cord blood	677CC or 677CT: *N* = 413 677TT: *N* = 50	Maternal 677TT genotype associated with lower DNAm in infant blood at candidate CpG sites in *NR3C1*, *DRD4*, *5-HTT*, *IGF2DMR*, *H19*, *KCNQ1OT1*, and *MTHFR* genes (*p* = 0.03)
Weiner et al. (2014) [36]	Genome-wide: Methyl Flash Methylated DNA Quantification Kit	Blood	677CC: *N* = 40 677TT: *N* = 40	677TT associated with significantly lower mean DNA methylation compared to 677CC (*p* = 0.0034)
Llanos et al. (2015) [37]	LINE-1: pyrosequencing	Female breast tissue	1298AA: *N* = 73 1298AC or 1298CC: *N* = 45	1298C allele associated with lower LINE-1 methylation (OR 0.96; 95% CI 0.93–0.98)
Song et al. (2016) [101]	Genome-wide: Illumina 450k array	Female breast tissue	Study population: *N* = 81	677T and 1298C alleles associated with differential methylation at 5 and 3 CpGs, respectively (unadjusted *p* value < 5.0 × 10^−5^). No sites reached significance at an adjusted *p* value < 0.05
Studies finding no association with DNAm				
Narayanan et al. (2004) [38]	Genome-wide: radiolabeled methyl group incorporation assay	Blood	677CC: *N* = 90 677CT: *N* = 84 677TT: *N* = 25 1298AA: *N* = 93 1298AC: *N* = 77 1298CC: *N* = 29	No altered DNAm in association with 677T or 1298C alleles
Jung et al. (2011) [102]	Genome-wide: LC/MS	Blood	(Folic acid supplemented/placebo) 677CC: *N* = 36/40 677CT: *N* = 36/34 677TT: *N* = 33/37	No altered DNAm between the 3-year folic acid-supplemented (0.8 mg/day) group and placebo group and no difference in DNAm when stratified by the *MTHFR* 677 genotype
Gomes et al. (2012) [39]	Genome-wide: IMDQ kit	Blood	677CC: *N* = 72 677CT: *N* = 39 677TT: *N* = 12	No altered DNAm between *MTHFR* 677 genotype groups
Ono et al. (2012) [103]	Genome-wide: LUMA	Blood	677CC: *N* = 112 677CT or 677TT: *N* = 272 1298AA: *N* = 254 1298AC or 1298CC: *N* = 130	No altered DNAm in association with *MTHFR* 677 or 1298 variants No interaction between genome-wide DNAm, folate intake, and *MTHFR* 677 or 1298 variants
Hanks et al. (2013) [104]	Genome-wide: LC/MS Candidate sites (*N* = 7): pyrosequencing	Colon	677CC: *N* = 185 677CT: *N* = 119 677TT: *N* = 32	No difference in DNAm between *MTHFR* 677 genotype groups, even when accounting for folate biomarkers No significant difference in DNAm at *ESR1*, *MYOD1*, *IGF2*, *N33*, *MLH1*, *MGMT*, and *APC* genes by the genotype group
de Arruda et al. (2013) [40]	Genome-wide: IMDQ kit	Oral epithelial cells	677CC: *N* = 17 677CT: *N* = 19 677TT: *N* = 8	No difference in DNAm between *MTHFR* 677 genotype groups
Deroo et al. (2014) [105]	LINE-1: pyrosequencing	Blood	Study population: *N* = 646 women without breast cancer *N* = 294 with breast cancer	677 or 1298 genotypes not associated with altered LINE-1 DNAm in women without breast cancer
Louie et al. (2016) [106]	Candidate sites (*N* = 3): bisulfite sequencing	Sperm	677CC: *N* = 21 677CT: *N* = 19 677TT: *N* = 4	677 genotype not associated with altered DNAm at *MEST*, *H19*, or *IG-GTL2* imprinted differentially methylated regions
Wang et al. (2016) [41]	Meta-analysis	11 studies	677: *N* = 1147 1298: *N* = 1053	No altered DNAm associated with 677T and 1298C alleles
Studies finding association with DNAm only with interaction with altered OCM nutrient status				
Friso et al. (2002) [33]	Genome-wide: LC/MS	Blood	677CC: *N* = 187 677TT: *N* = 105	677TT associated with approximately half the mean level of mCytosine than in the 677CC group (*p* < 0.0001). This effect was driven by TT individuals with low-folate status
Shellnut et al. (2004) [107]	Genome-wide: radiolabeled methyl group incorporation assay and LC/MS	Blood	677CC: *N* = 22 677TT: *N* = 19	No significant difference in DNAm between 677TT and 677CC groups. In response to 7-week folaterepletion following 7-week folate depletion, significantly increased mean % change and raw change in DNAm in 677TT individuals (*p* = 0.04 and 0.03, respectively)
Friso et al. (2005) [35]	Genome-wide: LC/MS	Blood	677CC/1298AA: *N* = 19 677TT/1298AA: *N* = 72 677CC/1298CC: *N* = 42	In the presence of low folate, 1298AA associated with lower genome-wide DNAm compared to 1298AC or 1298CC genotypes (*p* = 0.0001 and *p* = 0.021, respectively), and 677TT/1298AA associated with lower DNAm compared to 677CC/1298AA (*p* < 0.05) and 677CC/1298CC (*p* < 0.0001). In 677TT/1298AA individuals, DNAm significantly reduced in low-folate vs high-folate individuals (*p* < 0.0001)
Axume et al. (2007) [108]	Genome-wide: cytosine extension assay	Blood	677CC: *N* = 14 677CT: *N* = 12 677TT: *N* = 17	677TT associated with lower DNAm compared to 677CC (*p* < 0.05) after 7-week folate restriction followed by 7-week folate repletion treatment
La Merrill et al. (2012) [109]	Genome-wide: LUMA	Blood (pregnant women)	677CC: *N* = 31 677CT or 677TT: *N* = 164 1298AA: *N* = 158 1298AC or 1298CC: *N* = 37	677T or 1298C alleles not associated with altered genome-wide DNAm, but vitamin B_6_ deficiency and presence of 677T allele associated with hypomethylation (*p* = 0.02)
Aarabi et al. (2015) [110]	Genome-wide: RRBS Candidate sites (*N* = 6): pyrosequencing	Sperm	677CC: *N* = 13 677CT or 677TT: *N* = 17	After 6 months of high-dose folic acid supplementation, there was significant reduction in methylation in intergenic regions in 677CC men, whereas 677CT or 677TT men had significantly reduced methylation in promoters, exons, introns, and intergenic regions (*p* < 0.05)

*LUMA* luminometric methylation assay, *LINE-1* LINE-1 repetitive elements, *LC/MS* liquid-chromatography tandem mass spectrometry, *IMDQ* imprint methylated DNA quantification

^†^Sample size given for each *MTHFR* SNP was assessed in publication. If the sample size of specific genotypes is not present, it was not reported in publication. The combined *MTHFR* 677/1298 genotypes are specified when available; otherwise, these SNPs were assessed independently from one another in the same sample population

Several studies reviewed in Table 5 suggest that altered DNAm in association with *MTHFR* 677 and 1298 variants might only be present under limited folate conditions [33, 35]. The presence of folate stabilizes the variant MTHFR 677 enzyme [87], and adequate folate attenuates the effects of high-risk *MTHFR* 677TT genotype on increased homocysteine [88, 89]. Due to the retrospective nature of the study, we were unable to assess folate concentrations in the placenta or maternal blood and did not have complete information on maternal folic acid supplementation. Though folate status was unknown for the cases in this study, we assume that most of the women in our Canadian cohort were folate-replete due to folic acid fortification in cereal and grain, increased literacy around healthy pregnancies, and high uptake of gestational monitoring. Additionally, in a study of 368 pregnant women in Toronto, Canada, with similar demographics as our population in Vancouver, Plumptre et al. found that none of these women were folate deficient during pregnancy, even considering that 7% of women did not take folic acid supplements [90]. It is possible that in the presence of adequate folate levels, the activity of the variant MTHFR 677 or 1298 enzymes in the placentas of our study was not diminished enough to result in a compromised OCM and altered DNAm. Despite this potential limitation, investigating alterations in placental DNAm in association with *MTHFR* variants in a folate-replete population under the hypothesis that this may increase risk of pregnancy complications is still warranted. Fortification of grain products with folic acid has not entirely reduced the incidence of NTDs in folate-replete populations [91, 92]; in Canada, NTDs are the most common congenital abnormality [93]. Additionally, pathologies such as PE and IUGR are also present at a high frequency in folate-replete populations, and associations between PE and *MTHFR* have been observed in such populations [27], indicating a mechanism for association with pathology beyond low-folate/folic acid status.

In our study population, we found no significant association of NTDs, EOPE, LOPE, or nIUGR with high-risk 677TT or 1298CC placental genotypes, although there was a tendency to increased *MTHFR* 677TT in pregnancies affected by PE or IUGR as a whole (OR 2.53, *p* = 0.048). This trend is consistent with the literature noting an increased risk of PE in association with the 677T allele in both the maternal blood and the placenta [27, 29]. In a recent meta-analysis of 52 different studies, with a combined total of 7398 PE cases and 11,230 controls, Wu et al. identified a significantly increased risk of PE in association with the *MTHFR* 677T allele [28]. However, in 1103 cases and 988 controls, no association between the *MTHFR* 1298C allele and PE [28] was found. As for NTDs, our data was not suggestive of any association with fetal *MTHFR* 677TT or 1298CC genotype. Sample size limited our power to detect significant differences between study groups; however, few studies have investigated associations between placental/fetal *MTHFR* variants and PE/IUGR and NTD pathologies in the Canadian population post-folic acid fortification, and the main focus of the current research was to assess altered placental DNAm in association with high-risk *MTHFR* genotypes. Larger studies in NTDs have identified increased risk in association with the 677 variant [25], but there is inconsistent evidence for an association with the 1298CC genotype [24, 94].

Population stratification, the presence of systematic differences in allele frequencies between cases and controls, typically due to differences in ancestry, can be a limitation of genetic or epigenetic association studies. Specifically, false positive or negative results can be a consequence of failing to match study groups on this variable. To address this, we utilized a novel approach to assess population stratification in our study groups using three continuous variables of ancestry based on a MDS analysis of a panel of AIMs. This is similar to studies using MDS of genome-wide genotypes (i.e., from a SNP array or DNA sequencing) in study samples combined with ancestry reference populations to identify ancestry outliers, select homogeneous groups, or infer ancestry [70, 71, 95], and to studies that include principal components or MDS coordinates highly associated with ancestry in statistical models to correct for ancestry [96, 97]. Other potential confounding factors for our study, such as maternal smoking status, diet, and medications taken during pregnancy, were not well documented in all cases included in this study and thus could have resulted in heterogeneity between study groups that we were unable to account for in statistical modeling.

Currently, the evidence supporting the relationship between *MTHFR* 677 or 1298 variant and pathology or altered DNAm is not conclusive enough for physicians to support implementing *MTHFR* genetic testing as a clinical practice [98]. Despite this, *MTHFR* genotyping is available from 50 certified labs in the USA [98], and testing is widely promoted in the naturopathic field, where patients are told that a “faulty genotype” may explain a list of symptoms and diseases including “anxiousness, adrenal fatigue, brain fog, cervical dysplasia, increased risk of many cancers, low thyroid, leaky gut, high blood pressure, heart attacks, stroke, Alzheimer’s disease, diabetes, and miscarriages” (https://sciencebasedmedicine.org/dubious-mthfr-genetic-mutation-testing/). These patients are advised to take supplements containing “methyl folate” and “methyl B12” to increase methylation and decrease their risk of disease development (https://www.jillcarnahan.com/2013/05/12/mthfr-gene-mutation-whats-the-big-deal-about-methylation/). Our findings, coupled with variable results from other studies, suggest that these variants may not be of such strong concern in terms of DNAm, particularly in healthy individuals meeting folate requirements; however, studies with larger sample sizes are required to validate this. At the very least, the negative results from our study suggest that if these variants have an effect on placental and thereby newborn health in Canada, it may not be through altered DNA methylation.

## Conclusions

DNA methylation (DNAm) alterations have been proposed to be the link between *MTHFR* 677C>T and 1298A>C variants and increased risk of pregnancy complications. In this novel study of DNAm in human placentas of high-risk *MTHFR* 677TT and 1298CC individuals, we did not find evidence of altered DNAm associated with these genotypes in numerous measures of genome-wide and CpG site-specific methylation. We conclude that widespread changes in DNAm do not occur in the placentas of *MTHFR* 677 and 1298 variant carriers in our folate-replete population. Further studies with larger sample sizes and/or in populations that are folate deficient may support or refute our results. The results from this study suggest that factors other than alterations in DNAm may contribute to the previously reported association between high-risk *MTHFR* genotypes and pathology.

## Additional files

Additional file 1: **Table S1.** PCR and pyrosequencing conditions. (DOCX 22 kb)

Additional file 2: Methods. (DOCX 20 kb)

Additional file 3: **Table S2.** Global minor allele frequencies of 50 AIM SNPs used to assess ancestry. (DOCX 25 kb)

Additional file 4: **Figure S1.** Distribution of ancestry coordinates derived from MDS of 50 AIM SNP genotypes. (DOCX 166 kb)

Additional file 5: **Figure S2.** Distribution of *N* = 277 study samples along 3 MDS ancestry coordinates. (DOCX 138 kb)

Additional file 6: **Table S3.** *MTHFR* 677 and 1298 genotype counts and Hardy-Weinberg equilibrium. (DOCX 20 kb)

Additional file 7: **Figure S3.** Distribution of unadjusted *p*-values by CpG density between high-risk 677 or high-risk 1298 placentas compared to reference placentas. (DOCX 230 kb)

## Abbreviations

1kGP: 1000 Genomes Project
450k array: Illumina Infinium HumanMethylation450 BeadChip
AIMs: Ancestry-informative markers
CpG: Cytosine-guanine dinucleotide
DMR: Differentially methylated region
DNAm: DNA methylation
EOPE: Early-onset preeclampsia
FDR: False discovery rate
HWE: Hardy-Weinberg equilibrium
IUGR: Intrauterine growth restriction
LOPE: Late-onset preeclampsia
MDS: Multidimensional scaling
MTHFR: 5,10-Methylenetetrahydrofolate reductase
nIUGR: Normotensive intrauterine growth restriction
NTD: Neural tube defect
OCM: One-carbon metabolism
PE: Preeclampsia
rDNA: Ribosomal DNA
SNP: Single-nucleotide polymorphism
Δβ: Delta-beta
