# Supplementary material for: No evidence for association of MTHFR 677C>T and 1298A>C variants with placental DNA methylation
Source: Clin Epigenetics. 2018 Mar 13;10:34. doi: 10.1186/s13148-018-0468-1 (PMC5851070; doi:10.1186/s13148-018-0468-1)
Supplement: Supplementary file 1 — Table S1. PCR and pyrosequencing conditions. (DOCX 22 kb) [file 13148_2018_468_MOESM1_ESM.docx]

**Table S1. PCR and pyrosequencing conditions.**

|  | *MTHFR* 677 | *MTHFR* 1298 | rDNA | Alu | LINE-1 |
| --- | --- | --- | --- | --- | --- |
| Primer sequence (5’ to 3’) |  |  |  |  |  |
| Forward | 5Biosg/CTC AAA GAA AAG CTG CGT GAT | TCC AGC ATC ACT CAC TTT GTG AC | 5Biosg/GTT TTG GGG TTG ATT AGA GG | 5Biosg/TTT TTA TTA AAA ATA TAA AAA TTA GT | TTT TGA GTT AGG TGT GGG ATA TA |
| Reverse | TGT CAT CCC TAT TGG CAG GTT | 5Biosg/ctt tgg gga gct gaa gga cta cta | AAA ACC CAA CCT CTC CAA C | CCC AAA CTA AAA TAC AAT AA | 5Biosg/AAA ATC AAA AAA TTC CCT TTC |
| Sequencing | AAG CAC TTG AAG GAG AA | AACAAA GAC TTC AAA GAC AC | S1: GGG TTG ATT AGA GGG TT  S2: TTT TGG GGA TAG GTG T  S3: GGG GGA GGT ATA TTT TT | AAT AAC TAA AAT TAC AAA C | AGT TAG GTG TGG GAT ATA GT |
| PCR reaction (µL) |  |  |  |  |  |
| dH2O | 9.22 | 9.22 | 16.8 | 16.3 | 16.3 |
| 10x PCR buffer | 1.5 | 1.5 | 2.5 | 2.5 | 2.5 |
| 1.25 nM dNTPs | 2.4 | 2.4 | 4 | 4 | 4 |
| 10 µM F/R primers | 0.6 | 0.6 | 0.5/0.5 | 0.5/0.5 | 0.5/0.5 |
| 5 U/µL Taq DNA  polymerase | 0.18 | 0.18 | 0.2 | 0.2 | 0.2 |
| DNA | 50 ng | 50 ng | 15 ng (bsc) | 30 ng (bsc) | 30 ng (bsc) |
| PCR cycling conditions |  |  |  |  |  |
| Step 1 | 95˚ C for 05:00 | 95˚ C for 05:00 | 95˚ C for 15:00 | 95˚ C for 15:00 | 95˚ C for 15:00 |
| Step 2 | 95˚ C for 00:20 | 95˚ C for 00:20 | 95˚ C for 00:20 | 95˚ C for 01:30 | 95˚ C for 00:20 |
| Step 3 | 57˚ C for 00:20 | 60˚ C for 00:20 | 54˚ C for 00:20 | 49˚ C for 01:00 | 50˚ C for 00:20 |
| Step 4 | 72˚ C for 00:20 | 72˚ C for 00:20 | 72˚ C for 00:20 | 72˚ C for 01:20 | 72˚ C for 00:20 |
| Step 5 | Go to step 2, 49 times | Go to step 2, 49 times | Go to step 2, 40 times | Go to step 2, 39 times | Go to step 2, 44 times |
| Step 6 | 72˚ C for 05:00 | 72˚ C for 05:00 | 72˚ C for 05:00 | 72˚ C for 05:00 | 72˚ C for 05:00 |
| PCR product sequenced | 5 ul | 5 ul | 3 ul | 12 ul | 12 ul |

rDNA, ribosomal RNA genes; bsc, bisulfite converted
