# Supplementary material for: No evidence for association of MTHFR 677C>T and 1298A>C variants with placental DNA methylation
Source: Clin Epigenetics. 2018 Mar 13;10:34. doi: 10.1186/s13148-018-0468-1 (PMC5851070; doi:10.1186/s13148-018-0468-1)
Supplement: Supplementary file 2 — Methods. (DOCX 20 kb) [file 13148_2018_468_MOESM2_ESM.docx]

## Methods

### Population stratification

Ancestry can be a significant confounder in genetic association studies and is important to assess. While self–reported ethnicity is often used as a surrogate for genetic ancestry, it is not always available and may not always reflect genetic ancestry in admixed populations. In our study, maternal reported ethnicity was available for 67% of cases, with no information of paternal ethnicity. Due to the lack of complete information and the desire to assess fetal (placental) genetic ancestry rather than that of the mother, we chose to utilize a panel of 57 ancestry informative marker SNPs (AIMs)[1-3] to assess population stratification in our study. This panel was designed by Phillips et al.[1-3] to differentiate between European, South Asian, East Asian, and African ancestry. Rather than use these SNPs to categorize samples into discrete ancestry groups, we developed a method in which ancestry could be described as a continuous variable, which may better reflect admixture in our population from Vancovuer, Canada.

N=287 placental villus DNA samples were genotyped at 55 AIM SNPs (2 assays were not designable) using the Sequenom iPlex Gold platform by the Génome Québec Innovation Centre at McGill University, Montreal, Canada. Samples with a call rate of <0.9 were excluded, and subsequent SNPs with a call rate of <0.9 were also excluded. This left 53 SNPs and 277 samples for ancestry assessment. In addition to our study samples, 2157 individuals from African (N=661), East Asian (N=504), European (N=503), and South Asian (N=489) populations from the 1000 Genomes Project (1kGP) Phase III[4] were added as ancestry reference populations. We were able to download genotypes for 50/53 SNPs from the 1000 Genomes Browser (NCBI). In both datasets, genotypes at the 50 AIM SNPs were re-coded as 0, 1, or 2 based on the minor allele in our genotyped placentas, where 0 represents homozygous reference, 1 heterozygous and 2 homozygous alternative genotypes. A pairwise Euclidean distance matrix was calculated using these genotypes in the combined placental and 1kGP dataset. Classical multidimensional scaling (MDS) was applied to this distance matrix to represent it in *k*=3 dimensions, as these three dimensions clustered the 1kGP populations by ancestry and were significantly different between these 4 1kGP ancestry populations (**Additional File 4: Figure S1**). We extracted the values from the first 3 coordinates from the MDS for the 277 study samples and utilized them to describe ancestry as a continuous measure to assess population stratification in the genetic association study in this article (**Figure 1**).

### References

1. Phillips C, Salas A, Sanchez JJ, Fondevila M, Gomez-Tato A, Alvarez-Dios J, Calaza M, de Cal MC, Ballard D, Lareu MV, Carracedo A, SNPforID Consortium: **Inferring ancestral origin using a single multiplex assay of ancestry-informative marker SNPs.** Forensic Sci Int Genet 2007, **1**(3-4):273-280.

2. Fondevila M, Phillips C, Santos C, Freire Aradas A, Vallone PM, Butler JM, Lareu MV, Carracedo A: **Revision of the SNPforID 34-plex forensic ancestry test: Assay enhancements, standard reference sample genotypes and extended population studies.** Forensic Sci Int Genet 2013, **7**(1):63-74.

3. Phillips C, Freire Aradas A, Kriegel AK, Fondevila M, Bulbul O, Santos C, Serrulla Rech F, Perez Carceles MD, Carracedo A, Schneider PM, Lareu MV: **Eurasiaplex: a forensic SNP assay for differentiating European and South Asian ancestries.** Forensic Sci Int Genet 2013, **7**(3):359-366.

4. 1000 Genomes Project Consortium, Auton A, Brooks LD, Durbin RM, Garrison EP, Kang HM, Korbel JO, Marchini JL, McCarthy S, McVean GA, Abecasis GR: **A global reference for human genetic variation.** Nature 2015, **526**(7571):68-74.
