# Supplementary material for: No evidence for association of MTHFR 677C>T and 1298A>C variants with placental DNA methylation
Source: Clin Epigenetics. 2018 Mar 13;10:34. doi: 10.1186/s13148-018-0468-1 (PMC5851070; doi:10.1186/s13148-018-0468-1)
Supplement: Supplementary file 3 — Table S2. Global minor allele frequencies of 50 AIM SNPs used to assess ancestry. (DOCX 25 kb) [file 13148_2018_468_MOESM3_ESM.docx]

**Table S2. Global minor allele frequencies of 50 AIM SNPs used to assess ancestry.**

|  | AFR | EAS | EUR | SAS |
| --- | --- | --- | --- | --- |
| rs39897 | 0.197 | 0.336 | 0.545 | 0.225 |
| rs239031 | 0.683 | 0.043 | 0.012 | 0.004 |
| rs722098 | 0.158 | 0.457 | 0.813 | 0.646 |
| rs730570 | 0.241 | 0.238 | 0.841 | 0.539 |
| rs734482 | 0.058 | 0.038 | 0.545 | 0.236 |
| rs756913 | 0.076 | 0.004 | 0.500 | 0.146 |
| rs773658 | 0.633 | 0.025 | 0.004 | 0.000 |
| rs881929 | 0.356 | 0.885 | 0.388 | 0.145 |
| rs896788 | 0.321 | 0.637 | 0.180 | 0.495 |
| rs984038 | 0.449 | 0.402 | 0.392 | 0.698 |
| rs1024116 | 0.266 | 0.093 | 0.538 | 0.309 |
| rs1335873 | 0.921 | 0.344 | 0.289 | 0.228 |
| rs1363345 | 0.532 | 0.172 | 0.638 | 0.313 |
| rs1426654 | 0.074 | 0.012 | 0.997 | 0.685 |
| rs1498444 | 0.074 | 0.249 | 0.436 | 0.336 |
| rs1519654 | 0.082 | 0.000 | 0.500 | 0.157 |
| rs1544656 | 0.388 | 0.127 | 0.803 | 0.357 |
| rs1573020 | 0.477 | 0.045 | 0.000 | 0.009 |
| rs1785864 | 0.130 | 0.388 | 0.582 | 0.293 |
| rs1886510 | 0.095 | 0.156 | 0.525 | 0.182 |
| rs1941411 | 0.125 | 0.003 | 0.701 | 0.169 |
| rs1978806 | 0.513 | 0.000 | 0.004 | 0.012 |
| rs2026721 | 0.548 | 0.002 | 0.053 | 0.022 |
| rs2040411 | 0.170 | 0.699 | 0.331 | 0.528 |
| rs2065160 | 0.454 | 0.782 | 0.101 | 0.121 |
| rs2065982 | 0.077 | 0.705 | 0.055 | 0.224 |
| rs2156208 | 0.707 | 0.059 | 0.542 | 0.187 |
| rs2196051 | 0.033 | 0.001 | 0.705 | 0.189 |
| rs2227203 | 0.034 | 0.079 | 0.436 | 0.231 |
| rs2303798 | 0.687 | 0.204 | 0.014 | 0.185 |
| rs2472304 | 0.033 | 0.164 | 0.599 | 0.156 |
| rs2572307 | 0.547 | 0.000 | 0.004 | 0.000 |
| rs2814778 | 0.964 | 0.000 | 0.006 | 0.000 |
| rs2835133 | 0.609 | 0.299 | 0.059 | 0.153 |
| rs3785181 | 0.099 | 0.622 | 0.088 | 0.134 |
| rs3827760 | 0.003 | 0.873 | 0.011 | 0.013 |
| rs5997008 | 0.523 | 0.022 | 0.007 | 0.040 |
| rs6026972 | 0.073 | 0.045 | 0.588 | 0.272 |
| rs7354930 | 0.086 | 0.000 | 0.344 | 0.047 |
| rs7897550 | 0.067 | 0.410 | 0.278 | 0.263 |
| rs9487258 | 0.285 | 0.112 | 0.739 | 0.389 |
| rs9522149 | 0.057 | 0.012 | 0.763 | 0.213 |
| rs10008492 | 0.017 | 0.001 | 0.566 | 0.065 |
| rs10131666 | 0.181 | 0.139 | 0.605 | 0.327 |
| rs10141763 | 0.465 | 0.460 | 0.044 | 0.299 |
| rs10843344 | 0.021 | 0.125 | 0.340 | 0.239 |
| rs10962599 | 0.023 | 0.000 | 0.726 | 0.173 |
| rs12913832 | 0.028 | 0.002 | 0.636 | 0.071 |
| rs16891982 | 0.036 | 0.006 | 0.938 | 0.059 |
| rs17625895 | 0.054 | 0.004 | 0.482 | 0.112 |

Data retrieved from four major global ancestry groups from the 1000 Genomes Project (Phase III). AFR, African; EAS, East Asian; EUR, European; SAS, South Asian
