# Supplementary material for: No evidence for association of MTHFR 677C>T and 1298A>C variants with placental DNA methylation
Source: Clin Epigenetics. 2018 Mar 13;10:34. doi: 10.1186/s13148-018-0468-1 (PMC5851070; doi:10.1186/s13148-018-0468-1)
Supplement: Supplementary file 4 — Figure S1. Distribution of ancestry coordinates derived from MDS of 50 AIM SNP genotypes. (DOCX 166 kb) [file 13148_2018_468_MOESM4_ESM.docx]

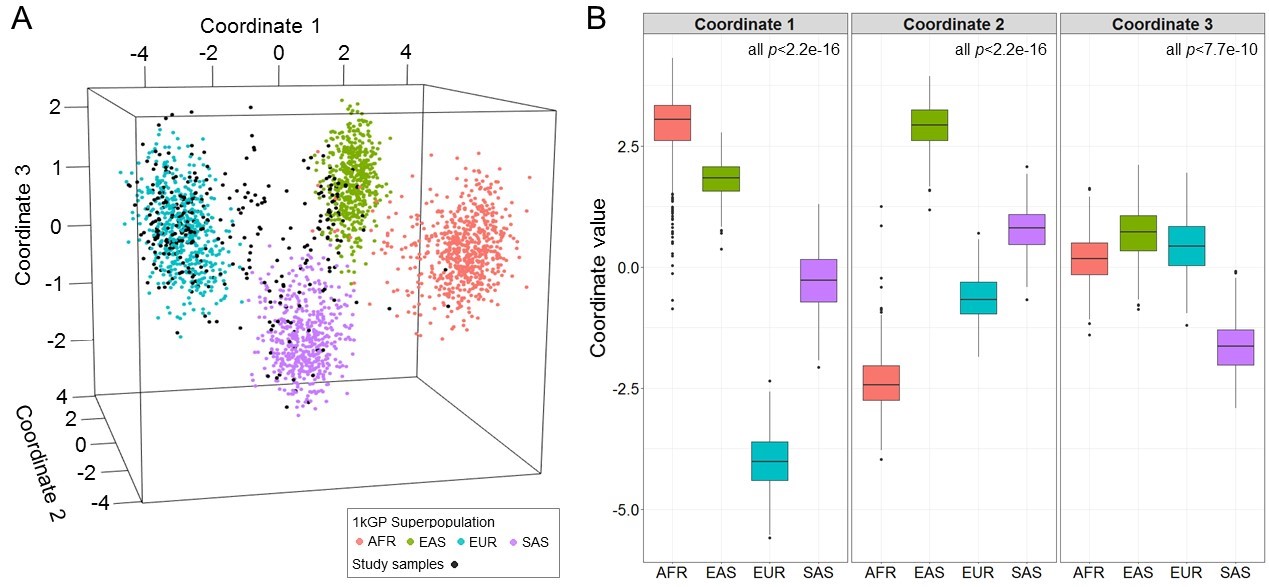
**Figure S1. Distribution of ancestry coordinates derived from MDS of 50 AIM SNP genotypes** in N=2157 1000 Genomes Project (1kGP) samples and N=278 placental samples from the current study. **A)** 3D plot of the 3 MDS coordinates shows that the 1kGP samples cluster by superpopulations, representing 4 major ancestry groups (AFR, N=661; EAS, N=504; EUR, N=503; SAS, N=489). Placental study samples (black) cluster with the EUR or EAS ancestries, as expected based on available maternal-reported ethnicity, with some samples clustering between groups indicating mixed ancestry. **B)** Boxplot of distributions of MDS ancestry values within each of the four 1kGP superpopulations. Within each coordinate, the distributions of values between the four populations are all significantly different from one another (pair-wise Kolmogrov-Smirnov tests, Bonferroni-corrected *p*-value<0.05). AFR: African; EAS: East Asian; EUR: European; SAS: South Asian.
