# Supplementary material for: No evidence for association of MTHFR 677C>T and 1298A>C variants with placental DNA methylation
Source: Clin Epigenetics. 2018 Mar 13;10:34. doi: 10.1186/s13148-018-0468-1 (PMC5851070; doi:10.1186/s13148-018-0468-1)
Supplement: Supplementary file 5 — Figure S2. Distribution of N = 277 study samples along 3 MDS ancestry coordinates. (DOCX 138 kb) [file 13148_2018_468_MOESM5_ESM.docx]

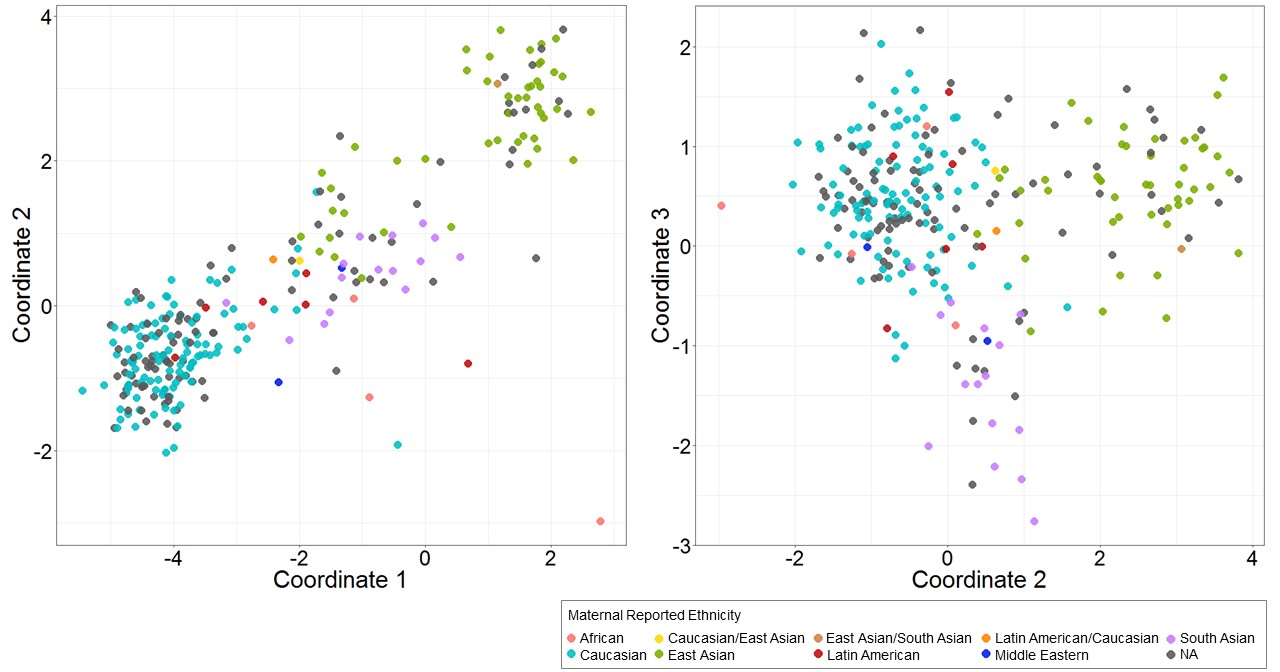


**Figure S2. Distribution of N=277 study samples along 3 MDS ancestry coordinates**. Samples are coloured by maternal reported ethnicity, where available. The 3 ancestry values are highly concordant with maternal reported ethnicity, with evidence for mixed ancestry of placental (fetal) DNA samples that cluster between major ancestry clusters.
