# Supplementary material for: No evidence for association of MTHFR 677C>T and 1298A>C variants with placental DNA methylation
Source: Clin Epigenetics. 2018 Mar 13;10:34. doi: 10.1186/s13148-018-0468-1 (PMC5851070; doi:10.1186/s13148-018-0468-1)
Supplement: Supplementary file 6 — Table S3. MTHFR 677 and 1298 genotype counts and Hardy-Weinberg equilibrium. (DOCX 20 kb) [file 13148_2018_468_MOESM6_ESM.docx]

**Table S3. *MTHFR* 677 and 1298 genotype counts and Hardy-Weinberg equilibrium in controls.**

|  | N | 677CC | 677CT | 677TT | HWE *p*-value | 1298AA | 1298AC | 1298CC | HWE *p*-value |
| --- | --- | --- | --- | --- | --- | --- | --- | --- | --- |
| Control | 179 | 88 | 81 | 10 | 0.5816 | 83 | 78 | 18 | 0.8893 |
| EOPE | 28 | 13 | 12 | 3 | -- | 15 | 13 | 0 | -- |
| LOPE | 20 | 13 | 4 | 3 | -- | 12 | 5 | 3 | -- |
| nIUGR | 21 | 9 | 9 | 3 | -- | 13 | 7 | 1 | -- |
| NTD | 55 | 25 | 25 | 5 | -- | 30 | 21 | 4 | -- |

*p­-*values calculated using an exact test for HWE in the control group. HWE, Hardy-Weinberg equilibrium; EOPE, early-onset preeclampsia; LOPE, late-onset preeclampsia; nIUGR, normotensive intrauterine growth restriction; NTD, neural tube defect
