# Supplementary material for: No evidence for association of MTHFR 677C>T and 1298A>C variants with placental DNA methylation
Source: Clin Epigenetics. 2018 Mar 13;10:34. doi: 10.1186/s13148-018-0468-1 (PMC5851070; doi:10.1186/s13148-018-0468-1)
Supplement: Supplementary file 7 — Figure S3. Distribution of unadjusted p-values by CpG density between high-risk 677 or high-risk 1298 placentas compared to reference placentas. (DOCX 230 kb) [file 13148_2018_468_MOESM7_ESM.docx]

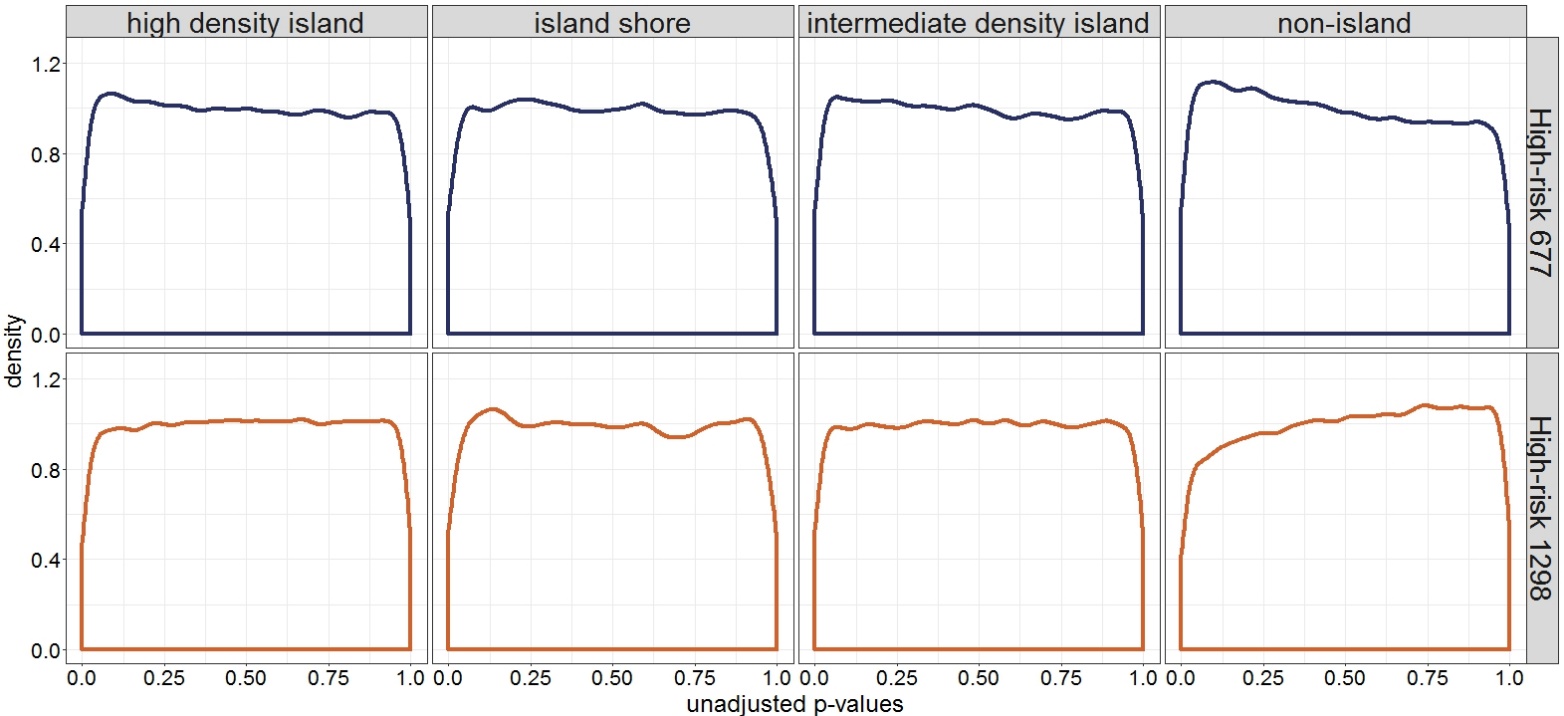
**Figure S3.** **Distribution of unadjusted *p*-values by CpG density between high-risk 677 or high-risk 1298 placentas compared to reference placentas**. N=442,355 CpGs on the 450k array were separated into four CpG density categories, and the linear model (*MTHFR* group as the main effect and fetal sex and gestational age included as covariates) was re-run within each CpG density category. No CpG density category (high density island, island shore, intermediate density island, non-island) showed a trend for differential methylation by either *MTHFR* high-risk genotype group.
